# Intrafraction displacement of prone versus supine prostate positioning monitored by real‐time electromagnetic tracking

**DOI:** 10.1120/jacmp.v14i2.4141

**Published:** 2013-03-04

**Authors:** Wayne M. Butler, Gregory S. Merrick, Joshua L. Reed, Brian C. Murray, Brian S. Kurko

**Affiliations:** ^1^ Schiffler Cancer Center Wheeling WV USA

**Keywords:** prostate, external beam radiation, Calypso, fiducials, intrafraction motion

## Abstract

Implanted radiofrequency transponders were used for real‐time monitoring of the intrafraction prostate displacement between patients in the prone position and the same patients in the supine position. Thirteen patients had three transponders implanted transperineally and were treated prone with a custom‐fitted thermoplastic immobilization device. After collecting data from the last fraction, patients were realigned in the supine position and the displacements of the transponders were monitored for 5–7 minutes. Fourier transforms were applied to the data from each patient to determine periodicity and its amplitude. To remove auto correlation from the stream of displacement data, the distribution of short‐term and long‐term velocity components were calculated from Poincaré plots of paired sequential vector displacements. The mean absolute displacement was significantly greater prone than supine in the superior–inferior (SI) plane (1.2±0.6mm vs. 0.6±0.4mm, p=0.015), but not for the lateral or anterior–posterior (AP) planes. Displacements were least in the lateral direction. Fourier analyses showed the amplitude of respiratory oscillations was much greater for the SI and AP planes in the prone versus the supine position. Analysis of Poincaré plots confirmed greater short‐term variance in the prone position, but no difference in the long‐term variance. The centroid of the implanted transponders was offset from the treatment isocenter by > 5 mm for 1.9% of the time versus 0.8% of the time for supine. These results confirmed significantly greater net intrafraction prostate displacement of patients in the prone position than in the supine position, but most of the difference was due to respiration‐induced motion that was most pronounced in the SI and AP directions. Because the respiratory motion remained within the action threshold and also within our 5 mm treatment planning margins, there is no compelling reason to choose one treatment position over the other.

PACS number: 87.50.st

## I. INTRODUCTION

The sharp dose gradients characteristic of intensity‐modulated radiation therapy (IMRT) require close attention to positioning and suggest that a mobile target should be tracked throughout treatment to deliver the planned dose to the target volume and planned sparing of organs at risk. Implanted gold fiducials may provide intrafraction prostate position information when imaged during the initial segment of each of five daily treatment beams.^(^ ^1^ ^)^ However, the Calypso 4D localization system (Calypso Medical Technologies, Seattle, WA) enables real‐time monitoring without the use of ionizing radiation, of implanted Beacon transponders (Calypso Medical Technologies). These transponders, queried at 10 Hz throughout the course of treatment, provide a very dense dataset of positional information.

Zelefsky et al.^(^ ^2^ ^)^ and McLaughlin et al.^(^ ^3^ ^)^ demonstrated significantly reduced dose to the rectum and bowel with patients in the prone position compared to supine. Although these publications pertained to 3D conformal radiotherapy, the dosimetric advantage of the prone position has been reported for IMRT,^(^ ^4^ ^)^ but there is a greater amount of prostate motion in the prone position compared to the supine position.^(^ ^5^ ^–^ ^7^ ^)^

Although patients prefer the more comfortable supine position,^(^ ^8^ ^)^ the geometric requirements of the Calypso system favor prone treatments. To effectively localize and continuously track the position of the radiofrequency transponders, their centroid must be < 19 cm from the 4D electromagnetic tracking array mounted over the patient on the treatment table. This distance limit increases by 6 cm to 25 cm from the array, if the goal is only to localize the patient isocenter for daily setup. About 15% of patients treated in the supine position exceed the continuous tracking distance limits due to anatomical factors such as a barrel‐shaped chest or protuberant abdomen.^(^ ^9^ ^)^ Because the prostate is somewhat posteriorly located, prone positioning reduces the distance between the transponders and the detector array by an amount sufficient to track prostate motion in almost all patients.

The purpose of this work was to evaluate and compare the intrafraction prostate displacement in terms of frequency and magnitude between our patients treated in the prone position and the same patients when repositioned in the supine position. There is abundant experience with real‐time tracking of patients in the supine position^(^ ^10^ ^)^ and reports of tracking behavior in patients treated prone,^(^ ^11^ ^)^ but there has been only two comparisons of tracking behavior in patients monitored in both positions.^(^ ^6^ ^,^ ^12^ ^)^ These two studies treated patients in the supine position and then repositioned the patients prone for a short interval of time. Our work not only intends to verify the results of Shah et al.^(^ ^12^ ^)^ and Kitamura et al.^(^ ^6^ ^)^ for patients planned and treated supine with patients planned and treated prone, but also to extend their work by separately evaluating long‐term and short‐term displacements by Poincaré analysis.

## II. MATERIALS AND METHODS

### A. Patient population

Thirteen patients consented to have their prostate displacements on the last day of treatment be compared in the prone and supine positions. The prescribed dose of 81 Gy was calculated to the 90% isodose line and delivered in 45 fractions of 1.8 Gy. The mean prostate volume was $49.9\pm 29.8{\text{cm}}^{2}$. The clinical target volume of prostate and seminal vesicles was expanded by 8 mm laterally and 5 mm in all directions to create the planning target volume. Patients were treated in the prone position with an empty bowel and bladder, and immobilized with a thermoplastic hip‐fix immobilizer custom‐fitted over the buttocks and abdomen at the time of the initial planning CT study. For each treatment fraction, the patient setup began with an initial alignment in the prone position of their tattooed skin marks to the lasers in the treatment room. The thermoplastic hip‐fix, marked during the planning CT scan of the patient, was then secured over the patient and aligned to the lasers in the room. Patient setup was completed by Calypso alignment of the centroid of three implanted Beacons with the accelerator isocenter. Calypso tracking was initiated after a cone‐beam CT scan — 180° arc — was performed on the patient to provide redundant verification of proper initial alignment. Typically, two minutes would elapse from initial Calypso alignment to the start of the first treatment beam and tracking.

### B. Electromagnetic beacons

Each patient had three passive radiofrequency Beacon transponders (8 mm long and 1.85 mm diameter) implanted transperineally to form a triangular array within the prostate under transrectal ultrasound guidance by one of the authors. Each Beacon in a set of three has a unique frequency response; therefore, each transponder vial, needle stylet, and needle hub is color‐coded with the intended position — prostate apex, right base, and left base. The accuracy of the Beacons has been verified to millimeter and even submillimeter accuracy both in phantoms and *in vivo* when compared to X‐ray and CT imaging.^(^ ^13^ ^,^ ^14^ ^)^

The Calypso system graphically displays the displacement of the centroid of Beacon transponders along each Cartesian axis. If the centroid position drifted from the machine isocenter beyond our threshold of 4 mm anterior–posterior (AP) or 5 mm superior–inferior (SI) or left/ right lateral, the therapist realigned the patient after completion of the current field and after installing the appropriate compensator for the next field. The patient was not realigned if there was a brief, transient spike in the centroid position followed by a rapid return to the zone of tolerance.

After treatment and tracking was completed in the prone position, the patient was moved into the supine position. To ensure the correct detection of the Beacons by Calypso, the physics staff manually changed the Beacon coordinate settings in the Calypso system by rotating the planning CT coordinates by 180° along the longitudinal axis. Using table movements, the therapists aligned the tattooed skin marks on the patient to the treatment room lasers. Supine setup was completed by using the Calypso system to align the centroid of the implanted Beacons with the accelerator isocenter. Tracking was then initiated and continued for 5 to 7 minutes with no thermoplastic immobilization and no treatment beams. Therapists were instructed to realign patients if the tracking position exceeded the usual treatment threshold. The data from this period of supine monitoring were compared with tracking data of the same duration for each patient during the initial period of that day's treatment in the prone position. Calypso tracking data for patients in the prone position included periods when radiation was being delivered, as well as periods during which the treatment beam was off, such as when the therapist entered the treatment room to change compensators.

### C. Data analysis

The raw Calypso data analyzed included the Beacon transponder coordinates in the three cardinal directions, x (right/left lateral), y (SI), and z (AP), as well as their respective timestamps during the tracking sessions. In the prone position, displacements are positive for movement in the right, superior, and posterior directions, and negative for movement in the left, inferior, and anterior directions. Data were recorded at a frequency of 10 Hz. The vector displacement, R, of the centroid from the intended, planned position was calculated for each data point from the equation:

(1) $$R=\sqrt{\Delta x^{2}+\Delta y^{2}+\Delta z^{2}}$$

The initial correlation of the transponder centroid to the treatment isocenter at t=0 normalized the values of Δx, Δy, and Δz to zero at that time.

Because of apparent respiratory‐induced periodic oscillations in the real‐time transponder displacement plots, we performed a Fourier transform on every displacement versus time graph. The resulting complex transform spectra were converted to plots of real relative amplitude vs. frequency to discern the characteristic frequency of the oscillations.

Because each measured displacement is strongly dependent on the value of its predecessor, these autocorrelations were obviated by analyzing Poincaré plots.^(^ ^15^ ^,^ ^16^ ^)^ For time‐dependent series, each point on these scatter plots have coordinates of the displacement at time, t, and at the subsequent time intervals, ($R_{n},R_{n+1}$), where n is within the sequence of measurements. Two calculated distributions were derived from Poincaré plots. The first, called the short‐term variance or short‐term velocity, is the perpendicular offset of each point from the diagonal identity line passing through the origin of the Poincaré plot. The offsets have units of cm/s because the two members of each coordinate pair were separated by 0.1 s. Points along the identity (diagonal) line have no offset from the preceding displacement. The greater the perpendicular distance a point is from the identity line, the greater is its positive or negative velocity. The short‐term variance is expected to be greater in patients with a pronounced respiratory‐induced prostate motion. The second distribution, long‐term variance or long‐term velocity, is found by constructing a line normal to the identity line and through the mean displacement. The perpendicular offset of each point relative to the mean normal line was calculated. Although the same data points as in the short‐term variance are used, the distances are calculated to a reference line orthogonal to the identity line. In the present study, those measured offsets represent largely aperiodic motions of the prostate arising from events such as bladder filling or patient movement.

### D. Statistics

Differences between prone and supine positioning were compared using a paired samples t‐test for comparing mean displacement data for the two positions. Distributions derived from Poincaré plots were compared between the prone and supine positions using the nonparametric Wilcoxon matched‐pairs signed‐rank test. The magnitude of breathing‐induced motion is proportional to the standard deviation. For example, if two patients are breathing at the same frequency and with mean amplitude of zero, the patient whose amplitude of sinusoidal waveform breathing is double that of the other will also have about twice the standard deviation. Statistical analyses were performed with PASW Statistics 17.0 (SPSS, Chicago, IL) or Stata 12.0 (StataCorp, College Station, TX). Significance was defined as a p‐value <0.05.

## III. RESULTS

**Figure 1:**
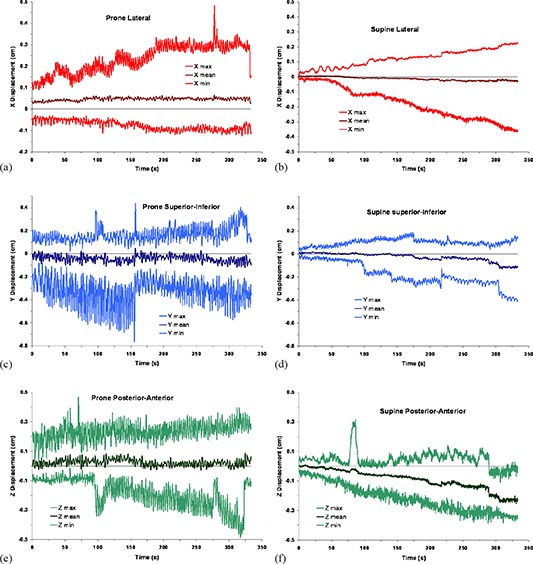
Prostate displacement versus time as determined by the centroid of the radiofrequency transducers: (a) patient prone, lateral displacements; (b) patient supine, lateral displacements; (c) patient prone, superior–inferior displacements; (d) patient supine, superior–inferior displacements; (e) patient prone, posterior–anterior displacements; (f) patient supine, posterior–anterior displacements. The middle curve on each graph is the mean displacement of the 13 patients in the study, and the upper and lower curves are the maximum and minimum, respectively.

The mean vector displacement of the prostate as determined by the position of the centroid of the implanted Calypso Beacons with time is shown in Fig. 1 for prone and supine positioning. The graphs are synchronized by the start time of tracking for the treatment fraction or the beginning of tracking in the supine position. Although prone treatment tracking continued for 7 to 10 minutes, the curves were truncated to the first 5½ minutes because of the shorter tracking times in the supine position. In each graph, the darker, central lines represent the mean displacement of the 13 patients at a particular time, and the lines above and below represent the individual maximum and minimum above and below the mean displacement value. In most of the graphs, a single patient defines the minimum and a different patient the maximum. However, the lower curve in Fig. 1(c) for superior–inferior displacements in the prone position represents two patients. At 155 seconds, the patient with large SI oscillations was realigned to isocenter during a compensator change because his excursions exceeded the 5 mm action threshold. With that patient reset to zero, a different patient began representing the greatest negative displacement from the mean.

**Figure 2:**
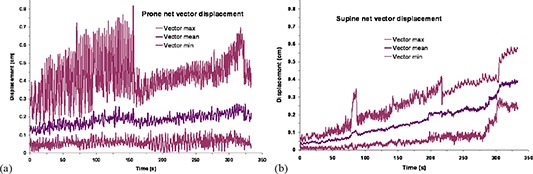
Net prostate displacement vs. time as calculated from the displacement of the centroid of the radiofrequency transducers in each Cartesian plane: (a) patient prone; (b) patient supine. The middle curve on each graph is the mean displacement of the 13 patients in the study, and the upper and lower curves are the maximum and minimum, respectively.

The net vector displacements of the centroid of the transponders are reported in Fig. 2. Because all net vector displacements must be ≥0, the large negative displacements of Fig. 1(c) dominate the maximum vector displacements for the first 155 seconds of Fig. 2(a). The mean net vector displacement curve showed only a slight trend toward increasing values from the beginning to the end of the observation period. A tendency for increased displacement with time was more pronounced for patients in the supine position (Fig. 2(b)), but this may be due to the absence of thermoplastic pelvic immobilization for patients in the supine orientation.

**Figure 3:**
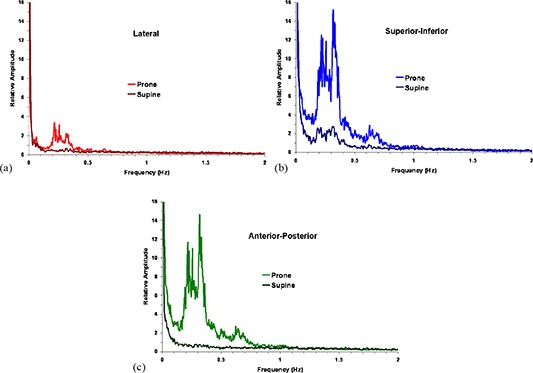
Composite Fourier transforms of displacement/time graphs into the relative amplitude/frequency domain for the study population: (a) left–right lateral; (b) superior–inferior; (c) anterior–posterior. The amplitude of the 10 Hz sampling frequency was defined as 100. In each graph, the amplitudes of respiratory oscillations in the range 0.2–0.4 Hz are much greater for patients in the prone position than when supine.

Composite Fourier transforms for the study population of the displacement/time graphs into the relative amplitude domain are shown for the two positions in Fig. 3. Fourier transforms for an individual patient show only 1 to 3 peaks at that patient's characteristic respiration frequency. In each composite graph, the amplitudes of respiratory oscillations in the range 0.2–0.4 Hz are much greater for patients in the prone position than when supine. In the latter position, respiratory oscillations are evident only in the SI plane, but at lower amplitude than in the prone position.

**Figure 4:**
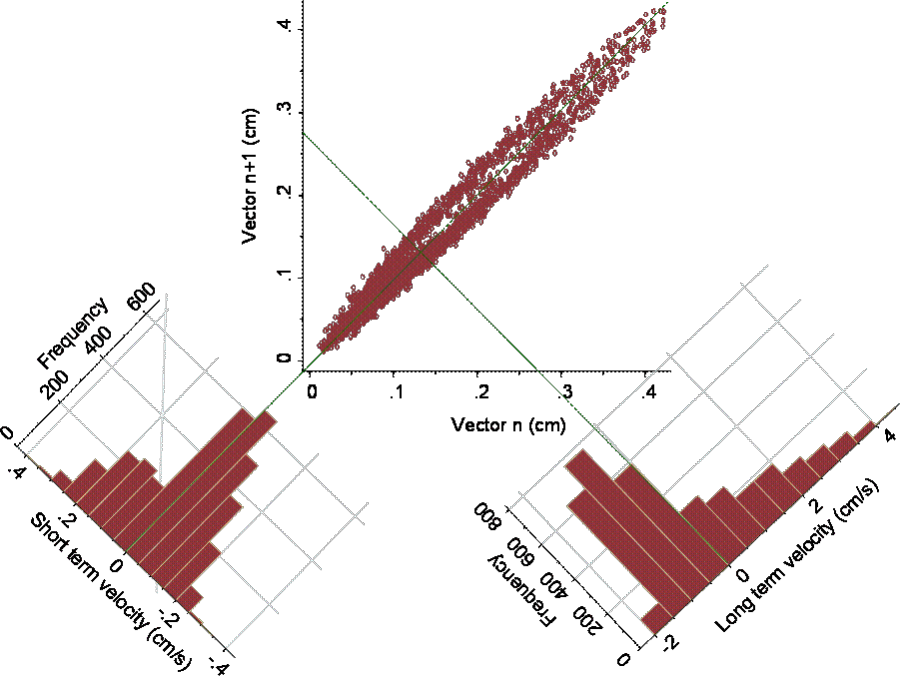
Poincaré plot and variance histograms of a patient in the prone position. Vector displacements were calculated for 3330 measurements of the centroid of three radiofrequency transponders in three cardinal planes. Each vector displacement in the acquisition sequence, n, was plotted against the next acquisition, n+1. A histogram of the short‐term variance or velocity relative to the diagonal identity line passing through the origin of the scatter plot is at the lower left. At the lower right is a histogram of the long‐term velocity, which measures the offset of each point relative to the line normal to the identity line and passing through the mean vector on the scatter plot. The scale of the latter histogram is about 10 times the scale of the former.

Although respiratory oscillations create obvious noise in prone displacement scans, the envelope of that noise was usually well within our action thresholds. To check whether those oscillations adversely affect the overall accuracy of treatments, Poincaré plots were analyzed. Figure 4 is a Poincaré plot of vector displacements at a given time as a function of the subsequent displacement in the next time interval, 0.1 s later. The calculated perpendicular distance to the diagonal identity line is a velocity related to short‐term variability. For each patient, another line normal to the identity line and passing through the mean vector displacement was constructed. The perpendicular offsets of points relative to this normal line were calculated as the long‐term variance.

**Figure 5:**
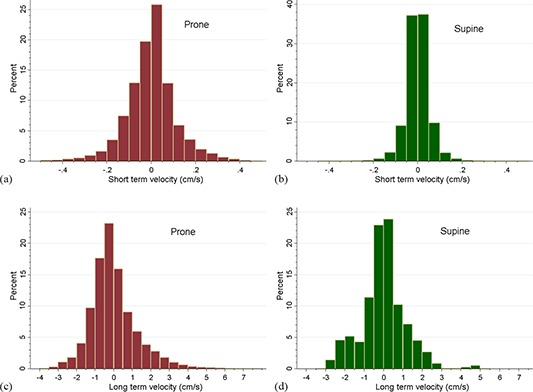
Histograms of the short‐term and long‐term variance for the 13 patients in the prone and supine positions: (a) short‐term velocity, prone; (b) short‐term velocity, supine; (c) long‐term velocity, prone; (d) long‐term velocity, supine. By the nonparametric Wilcoxon matched‐pairs signed‐rank test, the short‐term distributions (a) and (b) were significantly different for the two positions; however, the long‐term distributions (c) and (d) did not differ significantly.

Histograms of the short‐term and long‐term velocity distributions for the 13 patients in the prone and supine positions are show in Fig. 5. The nonparametric Wilcoxon matched‐pairs signed‐rank test found a significant difference between the two positions in the short‐term variance, p=<0.001, but no significant difference between the long‐term distributions, p=0.409. Therefore, both patient positions were equally effective in terms of net prostate motion during the course of treatment, but the prone position was noisier.

The mean displacements and the mean short‐term and long‐term variance in the prone and supine positions are listed in Table 1. The differences between the means were not clinically large; the net vector displacement was 1.84±0.74mm prone versus 1.24±0.65mm supine, but the difference between the means was significant by the paired samples t‐test. As with the Wilcoxon test of distributions described in the preceding paragraph, the mean short‐term variance was significantly different between the two positions, but not the long‐term variance via paired samples t‐test.

**Table 1:** Comparison of prostate displacements between patients in the prone vs. supine position. Means for the signed displacements in the three cardinal planes are in the first three lines of the table and absolute displacements in the next three. The short‐term and long‐term variances were calculated from data of Poincaré plots.

*Plane*	*Prone Displacement*		*Supine Displacement*		*p* ^a^
	*Mean*	*Std. Dev.*	*Mean*	*Std. Dev.*	
Lateral (mm)	0.46	±0.84	−0.09	±0.66	0.092
Superior–inferior (mm)	−0.42	±1.07	−0.14	±0.67	0.322
Anterior–posterior (mm)	0.23	±0.85	−0.62	±0.67	0.036
Absolute lateral (mm)	0.73	±0.57	0.45	±0.49	0.248
Absolute superior–inferior (mm)	1.15	±0.60	0.64	±0.37	0.015
Absolute anterior–posterior (mm)	0.92	±0.42	0.75	±0.61	0.339
Net vector (mm)	1.84	±0.74	1.24	±0.65	0.022
Short‐term variance (cm/s)	1.15	±0.99	0.64	±0.61	0.002
Long‐term variance (cm/s)	0.92	±0.79	0.83	±0.85	0.852

a Paired samples t‐test.

Using the standard deviation of displacements for each patient as a surrogate for oscillatory magnitude, the two treatment positions were compared in the study cohort by paired samples t‐tests in Table 2. By that criterion, the oscillatory magnitude in the SI and AP directions was significantly greater for patients in the prone position than in the supine position. This was confirmed by integrating the Fourier transforms of displacement/time graphs in the frequency range 0.01–2.0 Hz. The Fourier amplitudes in Table 2 are relative to the 10 Hz sampling frequency defined as 100. The relative amplitudes in each plane are in the same order and of the same magnitude as the standard deviations of the displacements.

**Table 2:** Mean standard deviation per patient of prostate displacements in each cardinal plane and the overall vector displacement for the 13 patients in the study compared with the relative Fourier transform amplitude in the region from 0.01 to 2.0 Hz.

					*Relative Fourier Transform Amplitude*			
					*Prone*		*Supine*	
*Plane*	*Prone Mean SD (mm)*	*Supine Mean SD (mm)*	*Correlation*	*p* ^a^	*Mean*	±SD	*Mean*	±SD
Lateral	0.25	0.29	−0.191	0.662	0.83	±0.75	0.84	±0.56
Superior–inferior	0.98	0.46	−0.061	0.002	3.22	±3.74	1.49	±1.21
Anterior–posterior	0.82	0.54	0.368	0.018	3.72	±3.07	1.85	±1.08
Net vector	0.81	0.63	0.237	0.234	0.83	±2.76	0.81	±1.56

a Paired samples t‐test.

Comparisons of the percent treatment duration that the centroid of the implanted transponders is offset from the treatment isocenter in 1 mm increments are listed in Table 3 for the three cardinal directions and for the overall displacement vector. The distributions of displacements were significantly different in all but the AP direction. In all instances, the percentage of time spent in displacements in the lowest increment, < 1 mm, is greater when patients are in the supine position. For absolute displacements greater than 2 mm, there was negligible difference between prone and supine positioning in the lateral or AP directions.

**Table 3:** Comparison of prostate displacements between prone and supine positioning as a percentage of time for each cardinal plane and the overall vector.

*Plane*	*Absolute Displacement (mm)*	*Prone (Percentage Time)*	*Supine (Percentage Time)*	*p* ^a^	*Prone* – *Supine (Percentage Time)*
Lateral	<1	77.3	85.8	<0.001	−8.5
	1 – 2	17.6	8.8		8.8
	2 – 3	3.9	4.3		−0.4
	3 – 4	1.2	1.1		0.1
	4 – 5	0	0		0
Superior–inferior	<1	54.7	82.0	<0.001	−27.3
	1 – 2	29.3	12.8		16.5
	2 – 3	10.0	4.4		5.6
	3 – 4	4.5	0.7		3.8
	4 – 5	1.0	0.1		0.9
	>5	0.6	0		0.6
Anterior–posterior	<1	66.7	68.7	0.090	−2.0
	1 – 2	20.9	18.9		2.0
	2 – 3	10.4	9.2		1.2
	3 – 4	1.7	3.1		−1.4
	4 – 5	0.2	0		0.2
Net vector	<1	20.3	49.0	<0.001	−28.7
	1 – 2	49.4	28.8		20.6
	2 – 3	14.9	13.3		1.6
	3 – 4	8.6	7.6		1.0
	4 – 5	4.9	0.6		4.3
	5 – 6	1.3	0.8		0.5
	>6	0.6	0		0.6

a Chi‐square test based on ~ 3300 data points per patient.

**Figure 6:**
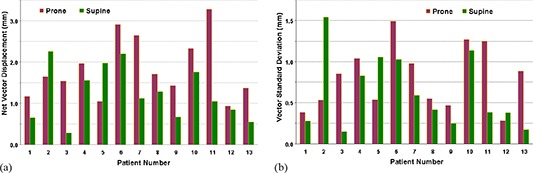
Net vector displacements (a) of the prostate for the 13 patients are correlated to systematic variations. Stratified by prone or supine positioning, only two patients (# 2 and # 5) had greater displacement in the supine position than prone. Mean vector standard deviation (b) of the vector displacements of the ~ 3,300 data points per patient. The SD of the vector displacements is correlated to random variance. Only three patients (#s 2, 5, and 12) had greater variance in the supine position than prone.

Not all 13 patients in the study group had smaller displacements in the supine position. Individual variability is graphed in Fig. 6. Two patients (numbers 2 and 5 in Fig. 6(a) had a greater net vector displacement in the supine position than in the prone, and three patients (2, 5, and 12 in Fig. 6(b) had a greater standard deviation in the supine position.

## IV. DISCUSSION

The mean vector displacement in the supine position was significantly less than that in the prone position (Table 1). Although only displacements in the superior–inferior plane were ever sufficient to initiate patient realignment (Table 3, 0.6% of the time), net vector displacements > 5 mm occurred 1.9% of the time. Our results agree with the report of Kitamura et al.,^(^ ^6^ ^)^ but their study's average prone position displacements for 10 patients in the SI and AP directions were considerably greater, while the standard deviations were similar to what we report here. The Kitamura study data were acquired during five 2‐minute sessions at 30 Hz, and the data were smoothed with a 30‐point rolling filter to reduce the noise. Kitamura and colleagues also noted a more pronounced respiratory oscillation for patients in the prone position which we illustrate in Figs. 1 and 2, comparing both positions. Vargas et al.^(^ ^17^ ^)^ applied automatic deformable segmentation of the prostate to seven patients in prone and supine positions to identify centroid motion in the sagittal plane using 4‐minute cine magnetic resonance imaging at 0.4 Hz. The Vargas study found mean displacements in the SI and AP planes similar to our results, but its mean SDs were considerably larger than the values we report in Table 2, and Vargas and colleagues found the SDs in the AP plane to be much greater than in the SI plane, compared to our finding of similar magnitude between the two planes. Important differences between the Vargas work and ours include the point that we found net prostate prone displacements to be virtually independent of time (Fig. 2), probably because of our intervention protocol, and the fact that our rigid prone pelvic immobilization apparently reduced the magnitude and SD of displacements compared to the lack of any immobilization in the Vargas and the Kitamura studies.

The Calypso system was used by Shah et al.^(^ ^12^ ^)^ to track prostate motion for patients in the prone position after a supine treatment. Although this study did not report mean and SD of displacements, it did illustrate the larger contribution of respiratory motion in prone tracking compared to supine. Shah and colleagues reported the percentage of time prostates underwent displacements at various thresholds were larger than ours. For net vector displacements > 5 mm, they found 10.1% and 2.9% of the time for prone and supine, respectively, while our corresponding values were 1.9% and 0.8%. The differences may be due to our interventions and the constraint of a pelvis thermoplastic mold in the prone position.

In the supine position, regular breathing was observed to involve abdominal oscillations. When patients are constrained in the prone position with a thermoplastic mold, abdominal respiration is not possible. In addition, patients have their chest partially supported by a semirigid foam pillow. This pillow may help channel chest and diaphragm motion into the pelvis.

Although our decision to treat patients in the prone position was based on published dosimetric comparisons, those studies did not involve real‐time observation of prostate motion, but rather static imaging pre‐ and post‐treatment.^(^ ^2^ ^–^ ^4^ ^)^ The prostate was separated from the rectum by about 5 mm in the prone position. In the context of the present study, one could ask: Is that advantage compromised by respiratory‐related prostate motion that is greatly enhanced by prone positioning with thermoplastic immobilization? This study found the prostates of most patients remained closer to the treatment isocenter and experienced less respiratory noise when in the supine position, but the magnitude of the difference was not sufficient to unequivocally choose one over the other. In the prone position, the greatest prostate displacements were in the superior–inferior direction and exceeded 5 mm only 0.6% of the time. Displacements in the lateral and anterior–posterior directions did not exceed 5 mm — the AP planning margin and also the typical posterior prostate‐to‐rectum separation. Therefore, our tracking and intervention protocol maintains the prostate within the planning margins.

One weakness of this observational study is the bias inherent in evaluating prostate motions with patients in the supine position after their treatment in the prone position. We also we did not assess patient comfort in either treatment position. Formal analysis of random and systematic variation was not performed in this study because the only repeated analysis is for the same patient in a different position. In the paper by Vargas et al.,^(^ ^17^ ^)^ random variation was correlated to the standard deviations (as in our Table 2) and systematic variations to the vector means (as in our Table 1). Finally, we did not attempt to assess the dosimetric consequences of the long‐term or short‐term displacements.

In previous work,^(^ ^11^ ^,^ ^16^ ^)^ prone positioning was necessary for large patients when the Calypso tracking array cannot be placed close enough to the centroid of the Beacons for localization and continuous tracking. Our use of Poincaré plots to separate short‐term variance — mostly due to respiration — from the long‐term variance showed that respiration effects were significantly greater in the prone position, but that there was no difference in long‐term variance. Because the total amplitude of respiratory‐induced displacements was within our action threshold limits and also within our 5 mm planning margins, there is no compelling reason to adopt supine positioning.

## V. CONCLUSIONS

Treatment of patients in the prone position was shown to result in significantly greater net intrafraction prostate displacement than in the supine position, but most of the difference was due to respiration‐induced motion that was most pronounced in the SI and AP directions. The long‐term variance was no different in the two positions. Displacements were least in the lateral direction and greatest in the SI direction. For patients with large abdominal/pelvic girth where the supine position may preclude accurate detection and tracking of implanted radiofrequency transponders, treatment in the prone position is a suitable alternative.
